# Sorghum Phytochrome B Inhibits Flowering in Long Days by Activating Expression of *SbPRR37* and *SbGHD7,* Repressors of *SbEHD1*, *SbCN8* and *SbCN12*

**DOI:** 10.1371/journal.pone.0105352

**Published:** 2014-08-14

**Authors:** Shanshan Yang, Rebecca L. Murphy, Daryl T. Morishige, Patricia E. Klein, William L. Rooney, John E. Mullet

**Affiliations:** 1 Department of Biochemistry and Biophysics, Texas A&M University, College Station, Texas, United States of America; 2 Department of Horticultural Sciences and Institute for Plant Genomics and Biotechnology, Texas A&M University, College Station, Texas, United States of America; 3 Department of Soil and Crop Sciences, Texas A&M University, College Station, Texas, United States of America; Karlsruhe Institute of Technology, Germany

## Abstract

Light signaling by phytochrome B in long days inhibits flowering in sorghum by increasing expression of the long day floral repressors *PSEUDORESPONSE REGULATOR PROTEIN* (*SbPRR37, Ma1*) and *GRAIN NUMBER, PLANT HEIGHT AND HEADING DATE 7 (SbGHD7, Ma6)*. *SbPRR37* and *SbGHD7* RNA abundance peaks in the morning and in the evening of long days through coordinate regulation by light and output from the circadian clock. 58 M, a phytochrome B deficient (*phyB-1*, *ma3^R^*) genotype, flowered ∼60 days earlier than 100 M (*PHYB, Ma3*) in long days and ∼11 days earlier in short days. Populations derived from 58 M (*Ma1, ma3^R^, Ma5, ma6*) and R.07007 (*Ma1, Ma3, ma5, Ma6*) varied in flowering time due to QTL aligned to *PHYB/phyB-1* (*Ma3*), *Ma5*, and *GHD7/ghd7-1* (*Ma6*). *PHYC* was proposed as a candidate gene for *Ma5* based on alignment and allelic variation. *PHYB* and *Ma5* (*PHYC*) were epistatic to *Ma1* and *Ma6* and progeny recessive for either gene flowered early in long days. Light signaling mediated by PhyB was required for high expression of the floral repressors *SbPRR37* and *SbGHD7* during the evening of long days. In 100 M (*PHYB*) the floral activators *SbEHD1*, *SbCN8* and *SbCN12* were repressed in long days and de-repressed in short days. In 58 M (*phyB-1*) these genes were highly expressed in long and short days. Furthermore, *SbCN15*, the ortholog of rice Hd3a (FT), is expressed at low levels in 100 M but at high levels in 58 M (*phyB-1*) regardless of day length, indicating that PhyB regulation of *SbCN15* expression may modify flowering time in a photoperiod-insensitive manner.

## Introduction

Flowering time has a significant impact on plant adaptation to agro-ecological environments, biomass accumulation and grain yield [1]. Floral initiation is regulated by plant development, photoperiod, shading, temperature, nutrient status, and many other factors [2]–[5]. Signals from many input pathways are integrated in the shoot apical meristem (SAM) through regulation of the meristem identity genes *LEAFY (LFY)* and *APETALA1 (AP1)*, which are activated during transition of the SAM from a vegetative meristem to a floral meristem. Long day (LD) plants, such as *Arabidopsis*, flower earlier in LD compared to short days (SD). In contrast, SD plants, such as rice and sorghum, show delayed floral initiation under LD conditions. Photoperiod regulated flowering is mediated by light signaling from photoreceptors and output from the endogenous circadian clock consistent with external coincidence models of flowering time regulation [6]. Photoperiod sensitive *Sorghum bicolor* genotypes delay floral initiation when grown under LD conditions. Sorghum genotypes with reduced photoperiod sensitivity have been identified and used by breeders because they flower early and at similar times in both long and short days, enhancing grain production [7]. In contrast, bioenergy sorghum is highly photoperiod sensitive, flowering in long day environments only after an extended phase of vegetative growth, thereby increasing biomass accumulation and nitrogen use efficiency [1], [8].

Photoperiod regulated flowering requires perception of light and signaling by plant photoreceptors such as the red/far-red light sensing phytochromes (Phy), blue light/ultraviolet wavelength sensing cryptochromes (Cry), phototropins, and Zeitlupes [9], [10]. Phytochromes play an important role in flowering time regulation in most plants including rice [11], barley [12], and sorghum [13]. The sorghum genome encodes three phytochrome genes, *PHYA*, *PHYB* and *PHYC*. Quail et al. (1994) established a standard nomenclature for phytochrome where PHY corresponds to phytochrome apoproteins, while phytochrome or phy indicates presence of the holoprotein, the fully assembled chromoprotein with chromophore covalently attached to the apoprotein [14]. Since all phytochrome proteins referred in this study are presumed to be holoproteins, Phy is used to represent wild type holoprotein, while phy is used to represent mutant versions of the holoprotein. Inactivation of PhyB results in early flowering in long days [13]. Phytochromes are soluble chromoproteins that contain an N-terminal photosensory domain and a C-terminal dimerization moiety. There are three sub-domains in the N-terminal moiety: PAS (PER, ARNT and SIM), GAF (cGMP phosphodiesterase, adenylate cyclase, Fh1A) and PHY (phytochrome-specific GAF-related), which form a unique structure, the “light-sensing knot” [15]. The PAS/GAF domains transduce light signals and the C-terminal domain, consisting of two PAS and HKRD (histidine-kinase-related domain), is responsible for dimerization and nuclear localization.

The central oscillators of the plant circadian clock are encoded by *TIMING OF CAB EXPRESSION 1 (TOC1), CIRCADIAN CLOCK ASSOCIATED 1 (CCA1)* and *LATE ELONGATED HYPOCOTYL (LHY)* [16]. Rhythmic expression of these central oscillators modulates the expression of *GIGANTEA (GI)*, an output gene of the circadian clock. GI, in concert with other factors, activates expression of *CONSTANS (CO)*, a zinc-finger transcription factor that plays an essential role in photoperiod regulation of flowering time in Arabidopsis [17], rice [18] and sorghum [19]. In *Arabidopsis*, CO is stabilized and accumulates during the evening of long days through the action of Cry1, Cry2 and PhyA, where it activates expression of *FT* and flowering. In SD, CO is not stabilized during the evening because *CO* expression occurs in darkness [20]. FT is produced in leaves and translocated to the SAM where it binds to FD. In Arabidopsis, FT together with *SUPPRESSOR OF OVEREXPRESSION OF CONSTANS (SOC1)*, promotes expression of meristem identity gene *LFY* and *AP1*, leading to floral transition [20].

The core of photoperiod regulatory pathway *GI-CO-FT* is present in *Arabidopsis,* a LD plant, and the SD plants rice and sorghum. In rice, *OsGI*, *HEADING DATE 1 (Hd1)*, and *HEADING DATE 3a (Hd3a)* are orthologs of *GI*, *CO*, and *FT*, respectively [21]. Hd1 (*OsCO*) delays flowering time in LD in rice and activates flowering in SD. In addition, Itoh et al. [22] identified a pair of genes in rice, *EARLY HEADING DATE 1 (EHD1)* and *GRAIN NUMBER, PLANT HEIGHT AND HEADING DATE 7 (GHD7)* that regulate flowering in response to day length by modifying expression of *Hd3a* (florigen). *EHD1* activates *Hd3a* expression and induces floral transition. In contrast, *GHD7*, a homolog of wheat *VRN2* [23], represses flowering in LD by down-regulating *EHD1* and *Hd3a*. In maize, 25 FT-like homologs were identified and designated as *Zea mays CENTRORADIALIS* (*ZCN*) genes. *ZCN8* was identified as a source of florigen [24]. *SbCN8* (ortholog of *ZCN8*) and *SbCN12* (ortholog of *ZCN12*) have been proposed to encode florigens in sorghum [19], [25], [26]. In sorghum, CO activates flowering in SD by inducing expression of *SbEHD1*, *SbCN8* and *SbCN12,* whereas in LD, CO activity is inhibited by SbPRR37 [19].

More than 40 flowering time QTL have been identified in sorghum [27] and maturity loci *Ma1–Ma6*, modify photoperiod sensitivity [7], [28], [29]. Dominance at *Ma1–Ma6* delays floral initiation in long days. *Ma3* encodes phytochrome B, indicating that light signaling through this photoreceptor is required for photoperiod sensitive variation in flowering time [13]. *Ma6* was identified as *SbGHD7*, a repressor of flowering in long days [26]. In LD, SbGhd7 increases photoperiod sensitivity by inhibiting expression of the floral activators *SbEHD1*, *SbCN12* and *SbCN8*. *Ma1* was identified as *SbPRR37*, a floral repressor that acts in LD [25]. The orthologs of *SbPRR37* in wheat and barley, *PHOTOPERIOD 1* (*Ppd1, Ppd-H1, Ppd-D1a*) [30], [31] and rice *OsPRR37* [32], also modulate flowering time in response to photoperiod. In LD, SbPRR37 inhibits expression of *SbEHD1*, *SbCN12*, and *SbCN8,* resulting in repression of flowering [25]. Moreover, *SbPRR37* modulates photoperiod sensitivity and floral repression in an additive fashion together with *SbGHD7* [26]. Expression of *SbPRR37* and *SbGHD7* is regulated by the circadian clock and light, suggesting common upstream regulation [26].

The current study focused on elucidating how phytochrome B regulates flowering time in response to day-length in sorghum. We report that *PHYB* is required for light activation of *SbPRR37* and *SbGHD7* expression in the evening of long days, resulting in repression of *SbEHD1*, *SbCN12*, *SbCN8* and floral initiation.

## Materials and Methods

### Phenotypic analysis of sorghum flowering time

**Figure 1:**
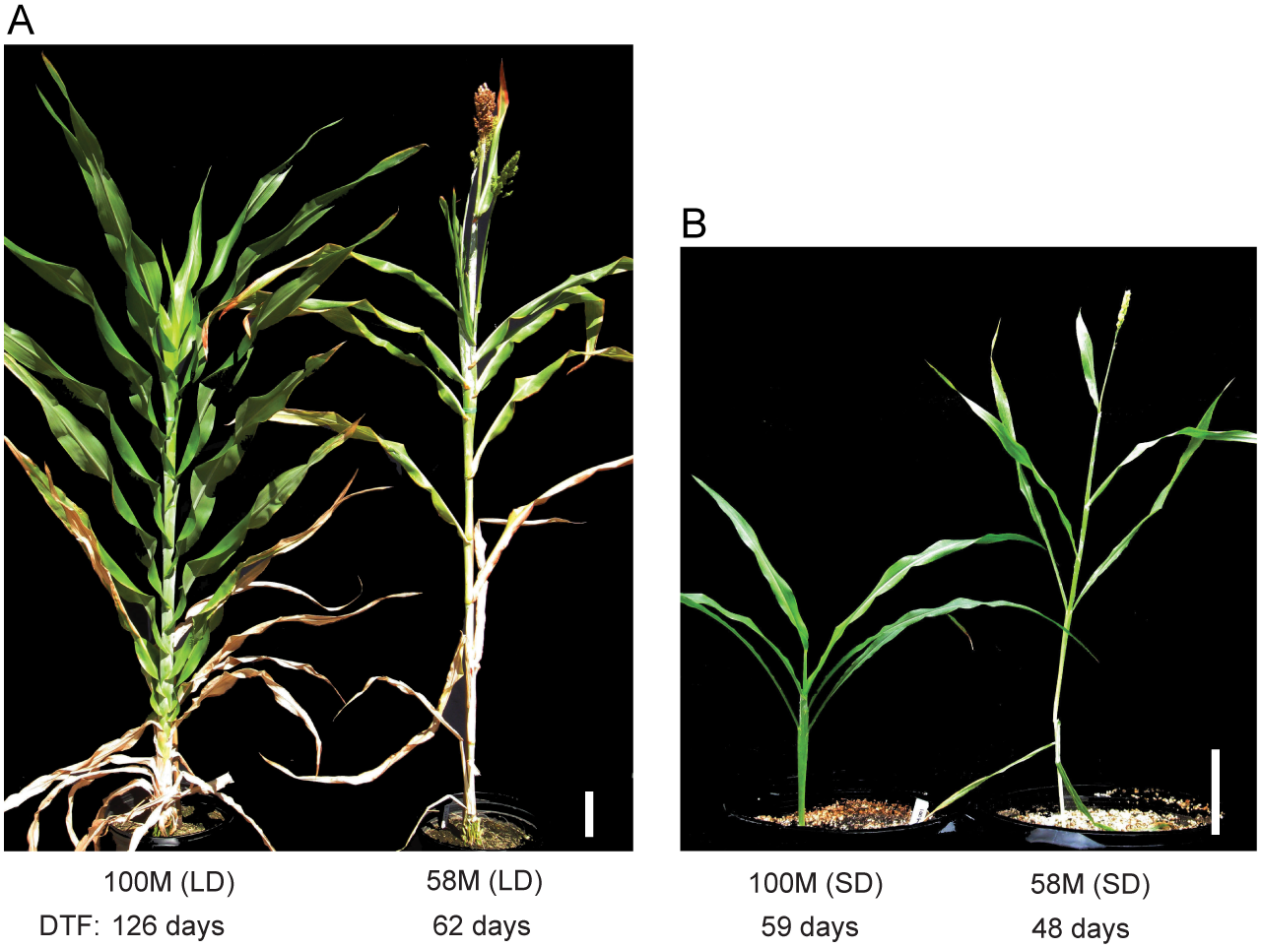
Photographs of the sorghum lines 100 M and 58 M for flowering time phenotype. (A) Photograph of 100 M (left) and 58 M (right) grown for 109 days in LD (14 h light/10 h dark). 100 M and 58 M flowered after 126 days and 62 days respectively. (B) Photograph of 100 M (left) and 58 M (right) grown in a greenhouse in SD for 53 days (10 h light/14 h dark). 100 M flowered after 59 days and 58 M flowered after 48 days. LD: long days. SD: short days. DTF = number of days to flowering time. Scale bar is 8.6 cm.

The maturity loci and flowering dates of all sorghum lines used in this study are listed in Table S1. To characterize the difference in flowering time between different genotypes and day-length, 100 M and 58 M were planted in Metro-Mix 200 (Sunshine MVP; Sun Gro Horticulture) and grown in a greenhouse in LD (14 h light/10 h dark) and SD (10 h light/14 h dark) conditions. Days to mid-anthesis were recorded and plants were photographed. 100 M plants (n = 5) and 58 M plants (n = 9) were grown in LD and phenotyped for days to anthesis (Figure 1A). The mean days to flowering for 100 M was 126 days (±4 days) and 62 days (±3 days) for 58 M, a significant difference in flowering times for these genotypes (p-value<<0.001, Welch two sample t-test). Under SD, 100 M plants (n = 7) and 58 M plants (n = 5) were used for analysis of flowering time (Figure 1B). The mean days to flowering for 100 M was 59 days (±4 days) and for 58 M, 48 days (±1 days), a significant differences in days to flowering (p-value<<0.001). To establish the interaction between PhyB and photoperiod, factorial ANOVA was run with photoperiod and PhyB alleles as factors. The significance of the effects of PhyB alleles, day-length and PhyB:day-length interaction were detected (p-value<<0.001). All statistics were run in R 3.1.0. The two-way interaction graphs were plotted using the “HH” package in R.

### Sequencing of *PHYB* alleles

To identify coding alleles in the *PHYB* gene, the full-length genomic sorghum *PHYB* genes from historical sorghum cultivars were amplified as three overlapping segments by PCR (Phusion High-Fidelity DNA polymerase, New England BioLabs, Inc). The amplified PCR products were cleaned and concentrated (QIAquick PCR Purification kit, QIAGEN). PCR products were separated by electrophoresis on 1% agarose gels. Specific PCR products were excised and purified (QIAquick Gel Extraction Kit, QIAGEN). The purified PCR products were sequenced using the BigDye Terminator v3.1 Cycle Sequencing Kit (Applied Biosystems) and the Applied Biosystems 3130xl Genetic Analyzer. All primers used for sequencing were designed using PrimerQuest^SM^ software (Integrated DNA Technologies, Inc) and are shown in Table S2. Sequencher v4.8 (Gene Codes) was used for sequence assembly and alignment with the BTx623 whole genome sequence of *Sorghum bicolor* (version 1.4) downloaded from Phytozome v8.0 (http://www.phytozome.net/). The SIFT (sorting intolerant from tolerant) program (http://sift.jcvi.org/) was utilized to predict whether an amino acid substitution affects protein function, based on the degree of conservation of amino acid residues in sequence alignments derived from closely related sequences.

### QTL analysis of *PHYB* action

The sorghum cultivar 58 M (*Ma1Ma2ma3^R^Ma4Ma5ma6*) was crossed to R.07007 (*Ma1ma2Ma3Ma4ma5Ma6*) to generate a population for QTL analysis. F1 generation plants were self-pollinated to produce F2 populations from which F3 populations were derived by self pollination. F2 and F3 populations were planted in the greenhouse and grown under long day conditions (14 h light/10 h dark). Days to mid-anthesis of panicles of plants from the F2 and F3 populations were recorded. The median, standard error, and range of Days to Flowering and the number of plants of each genotype analyzed from the F2 and F3 populations are shown in Table3. For analysis of epistatic interaction, three-way ANOVA was run to detect the effect of allelic variation in three maturity genes (*Ma3*, *Ma5* and *Ma6*) and three two-way interactions (*Ma3*:*Ma5*, *Ma3*:*Ma6*, *Ma5*:*Ma6*). The significance of the effects of single genes and genetic interactions were detected (p-value<<0.001). All statistics were run in R 3.1.0. The two-way interaction graph was plotted using the “HH” package in R.

For genotyping, genomic DNA of 86 F2 individuals and 132 F3 individuals was extracted from leaf tissue using the FastDNA Spin Kit (MP Biomedicals). Template for sequencing on an Illumina GAIIx sequencer was generated following the standard Digital Genotyping (DG) protocol [33]. Genotypes of all individuals from both populations were identified. The genetic map was constructed using the Kosambi mapping function in MAPMAKER v3.0 with 285 markers from the F2 population and 653 markers from the F3 population. QTL were mapped using the genetic map and the Composite Interval Mapping (CIM) function in WinQTL Cartographer v2.5 [34]. Significant LOD thresholds for QTL detection were calculated based on experiment specific permutations with 1000 permutations and α = 0.05 [35].

### Gene Expression Assays

Sorghum genotypes 100 M and 58 M were planted and grown in a greenhouse under long day conditions (14 h light/10 h dark) for 32 days and then transferred to growth chambers under either LD (14 h light/10 h dark) or SD (10 h light/14 h dark) conditions for seven days for entrainment prior to collection of leaf tissue. In the growth chamber, daytime (lights on) temperature was set at 30°C with a light intensity of ∼300 µmol·s^−1^·m^−2^ and night (lights off) temperature was set at 23°C. Relative humidity was ∼50% throughout the experiment. At day 39, leaf segments from the top three expanded leaves from three individual plants of each genotype and treatment were collected every 3 hours through one 24 h light-dark cycle and 48 h of continuous light. The leaf tissues at each time point were subjected to total RNA extraction using TRI Reagent (MRC) with the protocol for samples with high levels of polysaccharides. RNA was further purified using the RNeasy Mini kit (QIAGEN), including removal of DNA contamination by on-column DNase I digestion before reverse transcription. RNA integrity was examined on 1% MOPS gels. First-strand cDNA synthesis was performed using the SuperScript III First-Strand Synthesis System (Invitrogen) with oligo dT and random hexamer primer mix. After first-strand cDNA synthesis, the reactions were diluted to 10 ng/µl of the initial total RNA. Gene-specific qPCR reactions were carried out using Power SYBR Green PCR Master Mix (Applied Biosystems). 18S rRNA was selected as the internal control reference and the reactions were performed using the TaqMan Universal PCR Master Mix Protocol with rRNA Probe (VIC Probe) and rRNA Forward/Reverse Primer. All reactions were run on the 7900HT Fast Real-Time PCR System with SDS v2.3 software (Applied Biosystems). The specificity of each gene specific primer set was validated by melting temperature curve analysis. Amplification efficiency of each primer sets was determined by the serial dilution method [36] (Table S3). Relative expression was determined by the comparative cycle threshold (ΔΔCt) method [36] with calibration from most highly expressed samples. The calculated primer efficiencies were used to adjust data for relative quantification by the efficiency correction method [37]. Each relative expression value was derived from an average of three technical replicates and three biological replicates. The individual expression data points presented as 2^−ΔCt^ [38]. The significance (p-values) of the difference in expression between genotypes were detected using Welch two sample t-test in R 3.1.0 based on three technical replicates and three biological replicates. P-values were calculated either for certain time points of the day or all time points of the day.

## Results

### *PHYB* alleles in diverse sorghum lines

Sorghum genotype 58 M, a photoperiod insensitive early flowering line, has the genotype *ma3^R^ma3^R^*, corresponding to the *phyB-1* allele [13]. This allele contains a frame shift mutation that results in a prematurely terminated PhyB lacking regions of the protein necessary for dimerization and biological activity. To confirm and extend prior analysis of *PHYB* diversity in sorghum, alleles from several sorghum lines that vary in photoperiod sensitivity were sequenced and compared. The coding sequence of *PHYB* from BTx623 and 100 M (both *Ma3*) was 7285 bp in length consisting of four exons encoding a protein with 1178 amino acid residues. *PHYB* sequences from R.07007, Hegari, Tx7000, BTx642, SC56, Shallu and BTx3197 were identical to BTx623 and 100 M (*Ma3*). The *PHYB* sequence from 58 M (*ma3^R^*), referred to as *phyB-1* (Table 1), contains a mutation that renders the gene inactive [13]. No coding mutations were identified in 90 M, a line that encodes the weak allele *ma3* [28]. IS3620C encodes a different allele, designated *phyB-2*, which differs from *PHYB* by one INDEL and two SNPs, resulting in one amino acid deletion and two amino acid substitutions (Table 1). The first substitution in *phyB-2* could alter function because it produces an Asp^308^Gly change in the GAF domain of PhyB. The SIFT prediction score of this Asp^308^Gly substitution is 0.1, indicating moderate intolerance.

**Table 1:** Sequence analysis of *PHYB* coding alleles in different sorghum lines.

	Exon 1	Exon 1	Exon 3	Exon 4	Sorghum Genotypes
Nucleotide Variation	CAC>…	A>G	A>.	C>G	
Protein Modification	His>…	Asp>Gly	Premature stop codon	Leu>Val	
Mutation Position (AA #)	31	308	1023	1113	
Alignment with PHYB in*Arabidopsis* (AA #)	32	293	1007	1096	
Phytochrome Domain		GAF(N)			
***PHYB*** ** (** ***Ma3*** ** or ** ***ma3*** **)**	**−**	**−**	**−**	**−**	**BTx623, 100** **M, 90** **M, R.07007, Hegari, Tx7000, BTx642, SC56, Shallu, BTx3197**
***phyB-1*** ** (** ***ma3^R^*** **)**	**−**	**−**	**+**	**−**	**58** **M**
***phyB-2***	**+**	**+**	**−**	**+**	**IS3620C**

### PhyB affects flowering time in LD and SD

The sorghum maturity standards, 100 M and 58 M, were constructed from Milo genotypes that contain alleles of *Ma1* and *Ma3* that modify flowering time [28]. The sorghum maturity standard 100 M is photoperiod sensitive with a maturity genotype *Ma1Ma2Ma3Ma4Ma5ma6* [26]. The genotype 58 M is photoperiod insensitive, flowers early in LD and SD, and has the genotype *Ma1Ma2ma3^R^Ma4Ma5ma6* [26]. Genotype 58 M contains null alleles of *Ma3* (*ma3^R^, phyB-1*) and *Ma6* (*ghd7-1)*. When grown in a greenhouse under 14 h LD during the summer, 58 M plants were spindly and flowered in ∼62 days (±3 days), whereas 100 M flowered in ∼126 days (±4 days) due to the repressing action of SbPRR37 (*Ma1*) (Figure 1A). This result confirmed that loss of PhyB activity in 58 M reduces the ability of *Ma1* to inhibit flowering in LD (p-value<<0.001) [13]. When grown in a greenhouse in 10 h SD during December–February at lower light intensity, 100 M flowered in ∼59 days (±4 days) while 58 M flowered in ∼48 days (±1 days) (Figure 1B). Therefore in sorghum, PhyB has a smaller but still significant effect on flowering time in SD (p-value<<0.001). The factorial ANOVA with photoperiod and *PHYB* alleles as factors indicated the effects of PhyB, day-length and PhyB:day-length interaction are all significant (p-value<<0.001) (Figure S1-A).

### *PHYB* is epistatic to *Ma1 (SbPRR37)* and *Ma6 (SbGHD7)*

**Figure 2:**
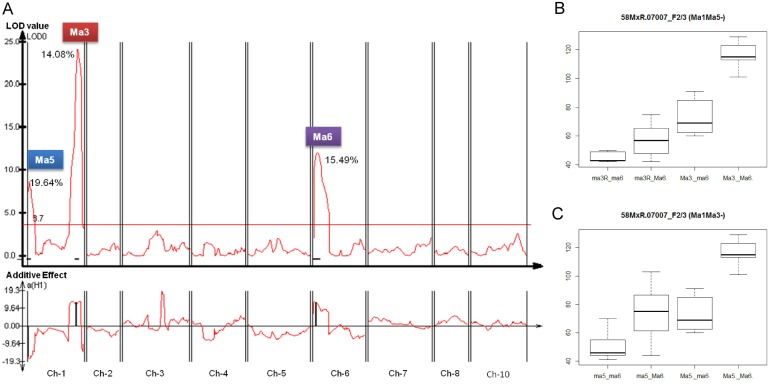
Flowering time QTL and analysis of epistasis in populations derived from 58MxR.07007. (A) Flowering time QTL labeled *Ma3, Ma5* and *Ma6*, were identified through analysis of flowering time variation in LD in the F2 population derived from 58MxR.07007. LOD values are shown on the Y-axis and sorghum chromosome numbers on the X-axis. The percent of the variance explained by each QTL is noted. The additive plot is shown in the lower portion of 2A where a positive value corresponds to alleles from R.07007 that delay flowering time. (B) Boxplot of flowering time distribution in the subset of the population with *Ma1Ma5-* genotypes but varying for alleles of *Ma3/ma3^R^* and *Ma6/ma6*. (C) Boxplot of flowering time distribution in the subset of the population having *Ma1Ma3*- genotypes but varying for *Ma5/ma5* and *Ma6/ma6.* Median values for flowering time are represented by horizontal lines within boxes.

In sorghum, *SbPRR37* (*Ma1*) and *SbGHD7* (*Ma6*) are primary determinants of photoperiod sensitivity in *Ma3* backgrounds acting in an additive fashion to inhibit flowering in LD [26]. Expression of both genes is induced by light, although the photoreceptor or photoreceptors that mediate light signaling were not known prior to the current study [25], [26]. To examine how *PHYB (Ma3)*, *SbPRR37* (*Ma1*), and *SbGHD7* (*Ma6*) co-regulate the timing of floral initiation, F2 and F3 populations were derived from a cross of R.07007 (*Ma1Ma3ma5Ma6*) and 58 M (*Ma1ma3^R^Ma5ma6*). These populations segregated for a wide range of flowering times (∼85 days) when planted in July and grown in a greenhouse in 14 h LD. Digital genotyping [33] was employed to generate DNA markers for genetic map construction. The genetic map spanned all of the ten sorghum chromosomes, although the long arms of SBI02 and SBI09 in its entirety were deficient in DNA markers. QTL analysis identified three significant QTL (LOD score>3.7) for days to anthesis in LD using the F2 population (n = 86), which together explained ∼50% of the phenotypic variance for flowering time (Figure 2A). The QTL with the highest LOD score (LOD = 24.2), spanned DNA on chromosome 1 from 60,402,909–61,604,749 bp which encompasses *PHYB* (chromosome_1∶60,915,677–60,917,553) (Table 2). Recessive *ma3^R^* alleles from 58 M associated with this QTL caused early flowering time phenotypes. The flowering time QTL on chromosome 6 spanning a physical interval from 203,707–1,716,581 bp (1 LOD interval) aligned with *SbGHD7* [26]. The recessive *ghd7-1* null allele from 58 M was associated with early flowering in LD. The third flowering time QTL near the proximal end of chromosome 1 (chromosome 1:6,139,583–9,077,991) had a LOD score of 8.7 and explained 19.6% percent of phenotype variance. This QTL was tentatively identified as *Ma5* because R.07007 was reported to be recessive for *Ma5*, a rare allele in sorghum [29]. No QTL aligned with *Ma1* as expected because both 58 M and R.07007 contain dominant alleles of *Ma1 (SbPRR37)*. The three flowering time QTL were also identified in the corresponding F3 population (data not shown).

**Table 2:** Information on flowering time QTL identified in the 58MxR.07007 F2 population.

QTL	Maturity Locus	Chromosome Number	Position (cM)^a^	LOD score	Physical Interval^b^	Additive Effect^c^	Dominant Effect^d^	R^2e^
1	*Ma5*	Ch_1	1.8	8.66	6139583–9077991	**−**17.09	18.19	0.1964
2	*Ma3*	Ch_1	99.4	24.21	60402909–61604749	12.55	16.09	0.1408
3	*Ma6*	Ch_6	7.2	12.09	203707–1716581	12.83	5.81	0.1549
Total								49.21%

a Position of likelihood peak (highest LOD score).

b Physical Interval: physical coordinate interval spanning 1 LOD interval across the likelihood peak.

c Additive Effect: A positive value means the delay of flowering time due to R.07007 allele. A negative value means the delay of flowering time due to 58 M allele.

d Dominant Effect: A positive value means dominance for the delay of flowering time.

e R^2^ (coefficient of determination): percentage of phenotypic variance explained by the QTL.

Plants from the F2/3 population are homozygous for *Ma1*, a repressor of flowering in LD, but varied in alleles of *Ma3, Ma5* and *Ma6*. Three-way ANOVA was used to analyze the effect of allelic variation in three maturity genes (*Ma3*, *Ma5* and *Ma6*) on flowering time, and three two-way interactions (*Ma3*:*Ma5*, *Ma3*:*Ma6*, *Ma5*:*Ma6*) showed that allelic variation of the three *Ma* genes and three two-way interactions were significant (p-values<<0.001). The three two-way interaction graphs between *Ma3*:*Ma5*, *Ma3*:*Ma6* and *Ma5*:*Ma6* are shown in Figure S1-B–D. Progeny with the genotypes *Ma3_Ma5_Ma6*_ and *Ma3_Ma5_ma6ma6* flowered later than genotypes that were homozygous recessive for *ma3^R^*, showing that *PHYB* is epistatic to the floral repressors encoded by *Ma1* and/or *Ma6* (Figure 2B; Figure S1-B,C). Progeny with the genotype *Ma3_Ma5_Ma6*_ (101–129 days) flowered later than plants with the genotype *Ma3_Ma5_ma6ma6* (60–91 days), consistent with increased floral repression due to *Ma6* in *Ma1* dominant backgrounds. The effect of *Ma6* was delay flowering with varying extents in different genetic backgrounds ranging from 14 days in *ma3^R^ma3^R^Ma5_*, ∼29 days in *Ma3_ma5ma5*, and ∼9 days in *ma3^R^ma3^R^ma5ma5*. Furthermore, it was noted that progeny lacking PhyB with a dominant *Ma6* allele showed a significant range of flowering times (42–75 days), suggesting that additional genes and/or environmental factors affect *Ma6* action in this genetic background (Figure 2B; Table 3). A similar wide range of flowering time (59 days) was observed among plants with the genotype *Ma3_ma5ma5Ma6*_ (Figure 2C; Table 3). In addition, plants with the genotype *Ma3___Ma5___Ma6___* flowered later in LD than plants with the genotypes *Ma3ma5ma5Ma6_* or *Ma3ma5ma5ma6ma6* (Figure 2C; Figure S1-D). This shows that *Ma5* is also required for late flowering in LD in *Ma1Ma3* backgrounds and that *Ma5* is epistatic to *Ma1* and *Ma6*. Plants with the genotype *ma3^R^ma3^R^Ma5_ma6ma6* and *Ma3_ma5ma5ma6ma6* flowered early and in a similar number of days as genotypes that are homozygous recessive for both *ma3^R^* and *ma5* (*ma3^R^ma3^R^ma5ma5ma6ma6*) indicating that the products of both *Ma3* and *Ma5* are required in LD for delayed flowering mediated by *Ma1* (*SbPRR37*).

**Table 3:** Flowering time of F2/F3 progeny from 58MxR.07007 in LD.

Genotype (All plants = *Ma1Ma1*)			Days to Flowering: median (±SE)	Days to Flowering: range	Number of plants
*Ma3_*	*Ma5_*	*Ma6_*	115 (±5)	101–129	42
*Ma3_*	*Ma5_*	*ma6ma6*	69 (±8)	60–91	19
*ma3^R^ma3^R^*	*Ma5_*	*Ma6_*	57 (±8)	42–75	15
*ma3^R^ma3^R^*	*Ma5_*	*ma6ma6*	43 (±2)	42–50	6
*Ma3_*	*ma5ma5*	*Ma6_*	75 (±12)	44–103	52
*Ma3_*	*ma5ma5*	*ma6ma6*	46 (±6)	41–70	30
*ma3^R^ma3^R^*	*ma5ma5*	*Ma6_*	53 (±6)	42–76	24
*ma3^R^ma3^R^*	*ma5ma5*	*ma6ma6*	44 (±6)	39–68	17

The requirement for both PhyB and the product of *Ma5* to observe delayed flowering in LD led us to examine the *Ma5* locus for candidate genes that might explain this interaction. The *Ma5* locus is located on SBI-01 and spans a large number of genes including several genes known to affect flowering time in other plants, including *AP1, CK2*, and *PHYC*. *PHYC* appeared to be the best candidate gene for *Ma5* because PhyC modifies flowering time in rice specifically in LD, similar to *Ma5* in sorghum [39], PhyB stabilizes PhyC, and PhyB:PhyC act as heterodimers in both Arabidopsis [40], [41] and rice [39], consistent with the co-dependence observed between *PHYB* and *Ma5* in this study. Comparison of *PHYC* sequences from BTx623 (*Ma5*), 100 M (*Ma5*), and R.07007 (*ma5*) revealed four differences in PhyC amino acid sequence between BTx623 and R.07007, and two differences between 58 M/100 M and R.07007 (Table 4). The latter amino acid variants occur in the PAS domain (Gly:Val) and HKRD domain (Glu:Asp) and SIFT analysis [42] indicated these changes could affect the function of PhyC. These results are consistent with *PHYC* as the candidate gene for *Ma5.* Further analysis is underway to test this assignment.

**Table 4:** Sequence analysis of *PHYC* coding alleles in different sorghum lines.

	Exon 1	Exon 1	Exon 1	Exon 2	Sorghum Genotypes
Nucleotide Variation	G>T	G>A	T>C	G>T	
Protein Modification	Gly>Val	Gly>Arg	Val>Ala	Glu>Asp	
Mutation Position (AA #)	124	162	190	922	
Alignment with PHYBin *Arabidopsis* (AA #)	160	198	226	954	
Phytochrome Domain	PAS(N)	PAS(N)	PAS-GAF Loop	HKRD(C)	
***PHYC-1*** ** (** ***Ma5*** **)**	**−**	**−**	**−**	**−**	**BTx623**
***PHYC-2***	**−**	**+**	**+**	**−**	**100** **M, 90** **M**
***phyC-1*** ** (** ***ma5*** **)**	**+**	**+**	**+**	**+**	**R.07007**

### PhyB modulates expression of *SbPRR37* and *SbGHD7* in long days

**Figure 3:**
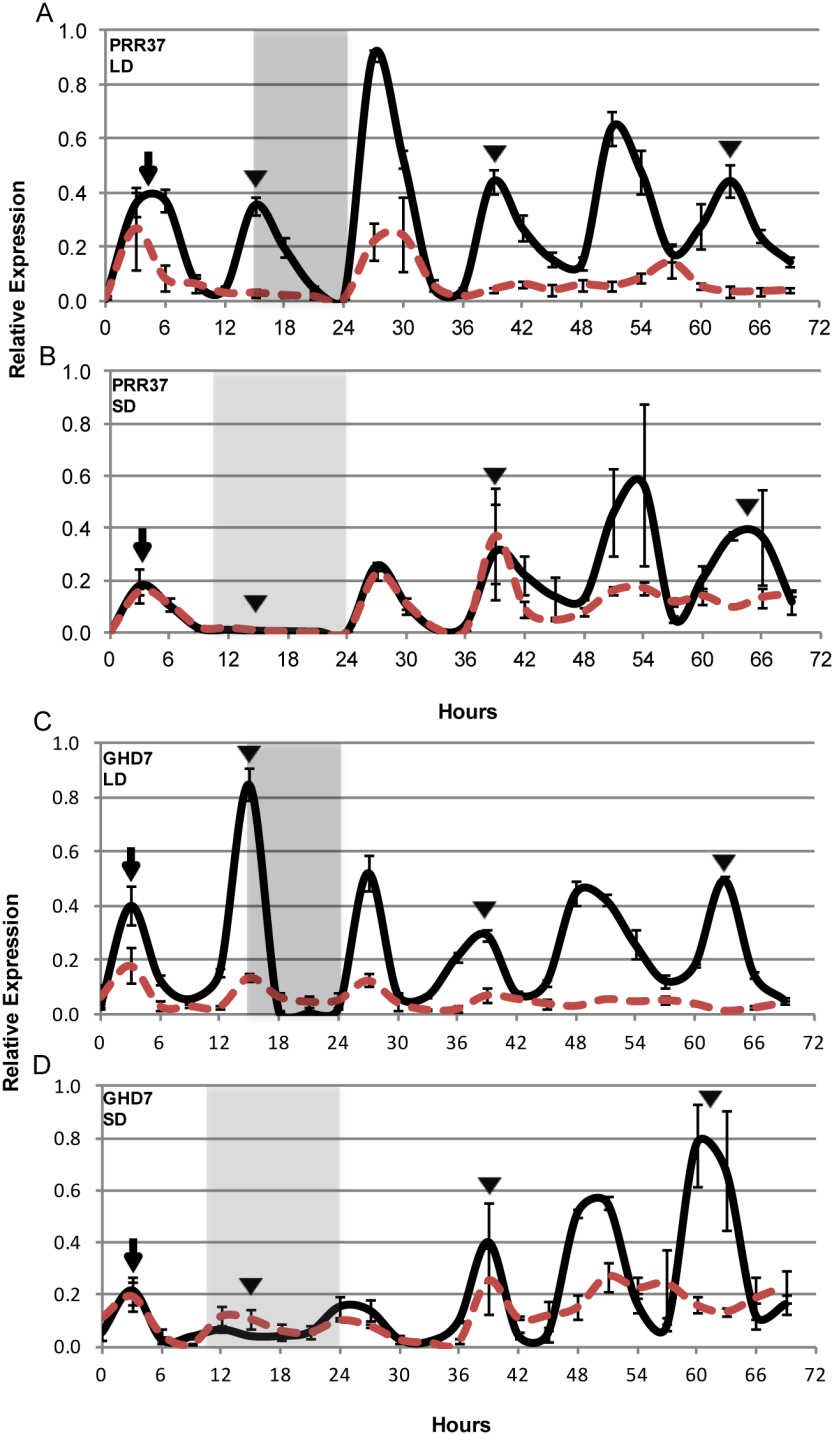
Relative expression of *SbPRR37* and *SbGHD7* in 100 M (*Ma3/PHYB*) and 58 M (*ma3^R^/phyB-1*) in LD and SD. 100 M (solid black line) and 58 M (dashed red line) plants were entrained LD (14 h light/10 h dark) or SD (10 h light/14 h dark) and sampled for one 24 h cycle, followed by 48 h in LL (continuous light and temperature). The grey background corresponds to time when plants are in darkness. Relative gene expression was determined every 3 hours by qRT-PCR. Arrows represent morning peaks of expression and arrowheads represent evening peaks of expression. (A) In LD, the second peak (arrowhead) of *SbPRR37* expression in the evening (∼15 h) is missing in the *phyB* deficient line, 58 M. (B) In SD, the second peak (arrowhead) of *SbPRR37* is absent in both 100 M and 58 M. (C) In LD, the second peak (arrowhead) of *SbGHD7* expression in the evening (∼15 h) is attenuated in 58 M. (D) In SD, the second peak of *SbGHD7* is attenuated in both 100 M and 58 M. Each data point of relative expression was based on data from three technical replicates and three biological replicates. Error bars indicate SEM.

Expression of *SbPRR37* and *SbGHD7* in leaves is regulated by light and gating by the circadian clock [25], [26]. The influence of PhyB on *SbPRR37* and *SbGHD7* expression was analyzed using 100 M (*PHYB*) and 58 M (*phyB-1*) plants grown for 32 days in LD then entrained for 7 days in LD or SD (Figure 3). Following entrainment, leaf samples were collected from plants for one 24 h LD or SD light-dark cycle, then from plants exposed to continuous light and temperature for an additional 48 h. In leaves of 100 M, *SbPRR37* and *SbGHD7* expression peaked in the morning (arrow) and evening (arrowhead) in LD as previously reported [25], [26] (Figure 3A/C, solid lines). *SbPRR37* and *SbGHD7* RNA abundance continued to oscillate with peaks in the morning and evening when 100 M plants were transferred to continuous light and temperature consistent with regulation by the circadian clock (Figure 3, 24–72 h). In leaves of 58 M in LD (Figure 3A/C, dashed red lines), *SbPRR37* and *SbGHD7* showed an increase in RNA abundance in the morning (arrow) but only a small increase in expression in the evening (arrowhead) compared to 100 M (Figure 3A, p-value<0.1; Figure 3C, p-value<0.05). These results indicate that PhyB is required for elevated evening expression of *SbPRR37* and *SbGHD7* in LD in 100 M.

When 100 M and 58 M plants were entrained and assayed in SD, the morning peak of *SbPRR37* expression was of similar amplitude in both genotypes and expression of *SbPRR37* was low during the evening (Figure 3B). Similarly, *SbGHD7* expression in SD was highest in the morning, reaching similar levels in 100 M and 58 M, and lower in the evening when compared to expression levels measured in LD (Figure 3D). These results indicate that in SD, PhyB has a limited effect on *SbPRR37* and *SbGHD7* expression. When 100 M plants entrained in SD were exposed to continuous light, the evening peak of *SbPRR37* and *SbGHD7* expression observed in LD reappeared on the first subjective day and expression levels were also elevated in the second subjective day (Figure 3B/D). In 58 M, the evening peak of *SbPRR37* and *SbGHD7* reappeared during the first subjective day, however overall expression was attenuated relative to 100 M during the second subjective day.

### PhyB modulates expression of *CO, Ehd1, SbCN8, SbCN12* and *SbCN15*

**Figure 4:**
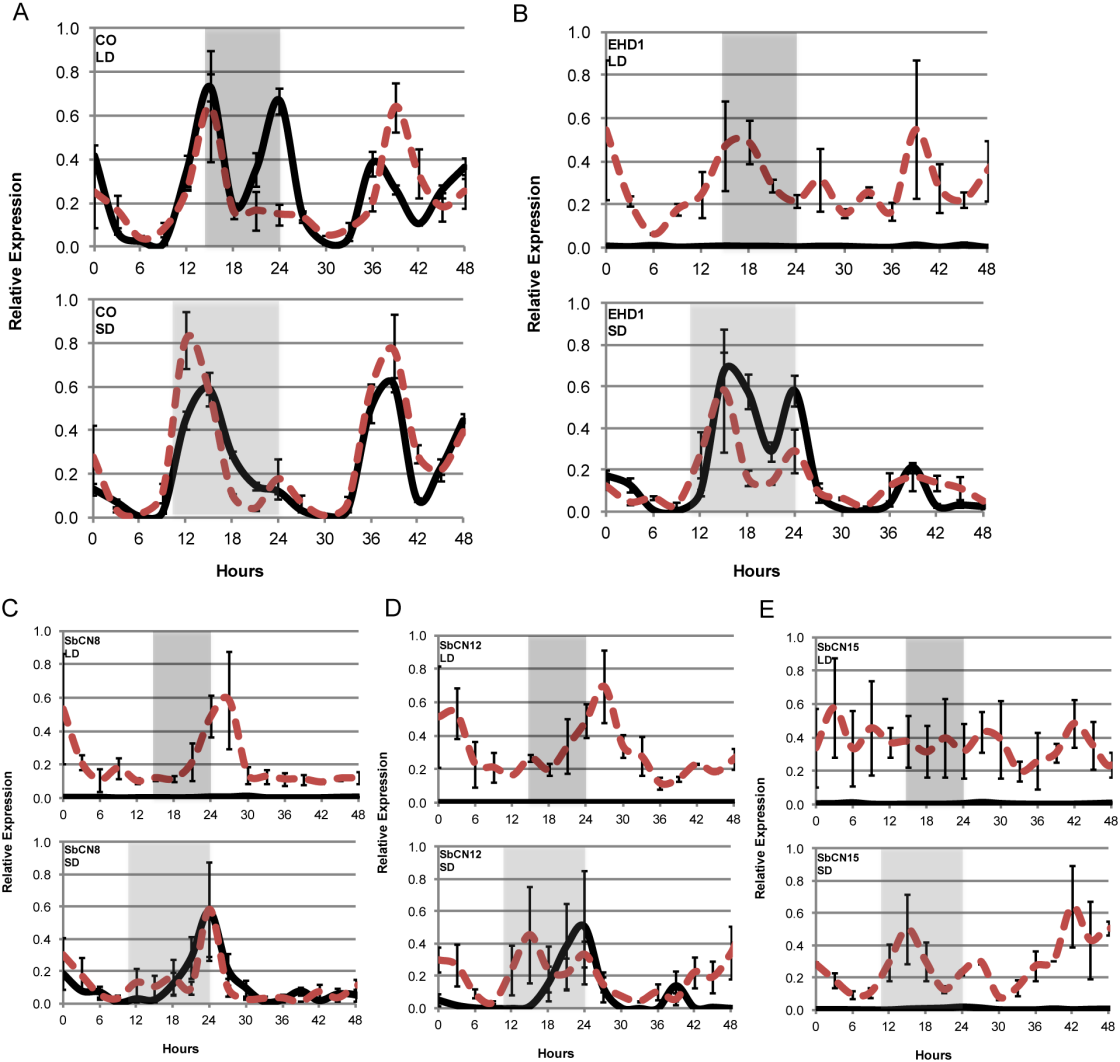
Expression of *SbCO*, *SbEhd1*, *SbCN8/12/15* in 100 M (*Ma3/PHYB*) and 58 M (*ma3^R^/phyB-1*) in LD and SD. Relative RNA levels in leaves of 100 M (solid black lines) and 58 M (dashed red lines) entrained and sampled in LD (14 h light/10 h dark) or SD (10 h light/14 h dark) for 24 h followed by 24 h in LL (continuous light and temperature). Relative expression levels were determined every 3 hours by qRT-PCR analysis. The gray shaded areas represent the dark periods. (A) *SbCO*, (B) *SbEHD1*, (C) *SbCN8*, (D) *SbCN12*, (E) *SbCN15*. Each data point of relative expression is based on three technical replicates and three biological replicates. Error bars indicate SEM.

In 100 M entrained to LD, the sorghum ortholog of *CONSTANS* (*SbCO*) shows peaks of expression at dawn (24 h) and in the evening (15 h) that are regulated by *SbPRR37*, the circadian clock, and day length [25]. In 58 M entrained and sampled in LD, the amplitude of the peak of *SbCO* expression at dawn (24 h) was reduced compared to 100 M (Figure 4A, p-value<0.05). The peak of *SbCO* expression at dawn was also reduced and of similar amplitude in plants entrained and sampled in SD (Figure 4A, lower). These results show that the peak of *SbCO* expression at dawn is dependent on PhyB, most likely because expression of *SbPRR37* in the evening of LD is dependent on PhyB (Figure 3A). In contrast, the evening peak (15 h) of *SbCO* expression was similar in both LD and SD in 100 M and 58 M indicating that PhyB does not significantly modulate *SbCO* expression at this time (15 h) of day.

*EHD1* is an activator of *Hd3a*, one of the florigens in rice [43]. The sorghum ortholog of *Hd3a* is *SbCN15*. Expression of *SbEHD1* increases when 100 M is transferred from LD to SD in parallel with increased expression of *SbCN8* (ortholog of *ZCN8* [24]) and *SbCN12* (ortholog of *ZCN12*) that have been proposed to encode florigens in sorghum [19], [25], [26]. *SbPRR37* and *SbGHD7* repress expression of *SbEHD1* in 100 M entrained in LD [25], [26]. Therefore *SbEHD1* expression in 58 M and 100 M was quantified and compared to determine if PhyB modulates *SbEHD1* expression. In LD, *SbEHD1* RNA abundance peaked in the evening and was up to ∼100-fold higher in 58 M relative to 100 M throughout the time course (Figure 4B, upper; Figure S2-A, p-value<<0.001). In SD, expression of *SbEHD1* was high in both genotypes and peaked during the night (Figure 4B, lower; Figure S2-A).

In 58 M entrained and analyzed in LD, expression of *SbCN8* (Figure 4C, upper) and *SbCN12* (Figure 4D, upper) peaked early in the morning and the relative abundance of RNA derived from these genes was elevated more than ∼100-fold relative to their levels in 100 M (Figure S2-B/C, p-values<<0.001). In SD, *SbCN8* (Figure 4C, lower) and *SbCN12* (Figure 4D, lower) expression was similar in both genotypes. Similarly, *SbCN15* (*Hd3a*) expression was increased up to ∼60-fold in 58 M compared to 100 M in LD and SD (Figure 4E; Figure S2-D, p-values<<0.001) at all time points assayed, indicating that PhyB mediated repression of *SbCN15* expression occurs regardless of photoperiod.

PhyB could be inducing *SbPRR37* and *SbGHD7* expression directly, and/or indirectly by altering output from the circadian clock. To determine if allelic variation in *PHYB* affected clock gene expression, *TOC1* and *LHY/CCA1*, the central oscillators, and *GI*, a mediator of clock output were examined (Figure S3). In LD and SD, *TOC1*, *LHY* and *GI* expression in 58 M and 100 M peaked at similar times and most of these genes showed similar amplitude of expression, although expression of GI was approximately 2-fold lower in 58 M. Although three biological replications at the indicated time points may not be sufficient to detect all biologically significant variation present, the small fold differences of circadian clock genes do not appear sufficient to explain the large variation in *SbPRR37* and *SbGHD7* expression observed in *Ma3* vs. *ma3^R^* backgrounds. *PHYB* and *PHYC* RNA levels were similar in 100 M and 58 M plants in LD and SD (data not shown).

## Discussion

Sorghum genotypes used for grain production are typically photoperiod insensitive and flower in 55–75 days when planted in April in locations such as College Station, Texas where day lengths increase during the early portion of the growing season. Early flowering in grain sorghum helps avoid adverse weather and insect pressure during the reproductive phase, thereby enhancing yield. In contrast, highly photoperiod sensitive energy sorghum genotypes planted in this same location will not initiate flowering for 175 days until mid-September when day lengths decrease to less than 12.2 h [1], [29]. Delayed flowering results in long duration of vegetative growth of energy sorghum, increasing biomass yield [8] and nitrogen use efficiency [8]. The importance of optimal flowering time for sorghum productivity led us to investigate the genetic and molecular basis of variation in this trait in sorghum.

**Figure 5:**
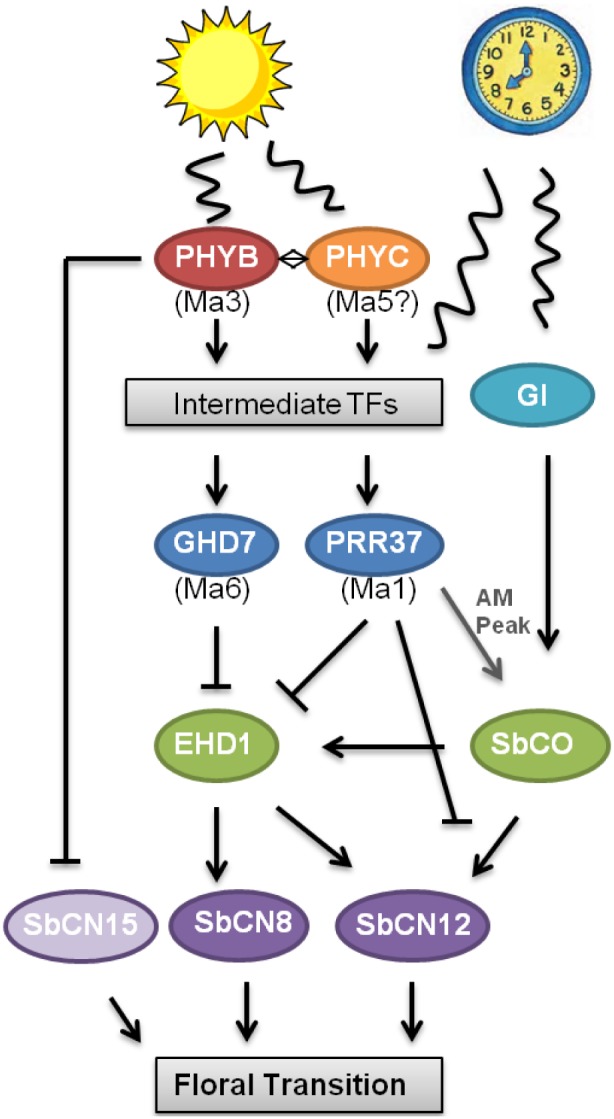
Model of the photoperiod flowering time pathway in sorghum. Phytochrome B (PhyB) is mediates light signaling that modulates flowering time in response to photoperiod in sorghum. In LD, PhyB up-regulates the expression of *PRR37* and *GHD7*, two central floral repressors, during the evening phase of LD but with minimal influence in SD. Induction at this time of day is also dependent on output from the circadian clock. PhyB may stabilize and interact with PhyC, a candidate gene for *Ma5* a locus that also contributes to photoperiod regulation of flowering time. SbPRR37 activates *SbCO* expression peaking at dawn. SbPRR37 and SbGhd7 repress expression of the floral inductors *SbEHD1, SbCN8, SbCN12* and *SbCN15*, leading to delayed flowering in long days. In SD or 58 M (*phyB-1*), expression of the floral repressors Sb*PRR37* and Sb*GHD7* is reduced which results in floral initiation once plants have satisfied other requirements for flowering. PhyB was found to mediate repression of *SbCN15* regardless of day length.

Variation of flowering time of sorghum germplasm grown in LD environments is caused principally by differences in photoperiod sensitivity, although shading, GA, temperature, length of the juvenile phase among other factors also affect this trait [7]. A model summarizing information about photoperiod regulation of flowering time in sorghum is shown in Figure 5. In LD, flowering is delayed in photoperiod sensitive sorghum by the additive action of the floral repressors, SbPRR37 (*Ma1*) and SbGhd7 (*Ma6*) [25], [26], [28], [29]. SbPRR37 and SbGhd7 repress expression of the grass specific floral activator, *SbEHD1*. In addition, SbPRR37 inhibits the activity of CO, another activator of flowering in sorghum [19]. The floral activators, SbEhd1 and SbCO, induce expression of *SbCN8* and *SbCN12*, the proposed sources of FT in sorghum. *SbCN15*, the ortholog of *Hd3a* and a source of florigen in rice [21], may also be a source of florigen in sorghum. The circadian clock is shown regulating expression of *SbGI, SbCO, SbPRR37* and *SbGHD7*, and light regulating expression of *SbGHD7* and *SbPRR37* as shown in previous studies [25], [26].

Photoperiod has minimal impact on flowering time in sorghum genotypes such as SM100 that encode null versions of *SbPRR37* and *SbGHD7* [25], [26]. Presence of functional alleles of either gene increases photoperiod sensitivity and a further delay in flowering is observed when both genes are present in dominant *Ma3Ma5* backgrounds. Expression of *SbPRR37* and *SbGHD7* is regulated by light and the circadian clock. Both genes show peaks of RNA abundance in the morning and again in the evening in LD and both peaks of RNA are attenuated in darkness. Importantly, the evening peak of expression is attenuated in SD when this phase occurs in darkness, indicating a requirement for light signaling during the evening to maintain sufficiently high levels expression of *SbPRR37* and *SbGHD7* to inhibit flowering. The morning and evening peaks of *SbPRR37* and *SbGHD7* expression observed in sorghum in LD is a pattern of expression first observed in photoperiod versions of this C4 grass. In Arabidopsis, *PRR7*, the ortholog of *SbPRR37*, shows a single peak of clock-regulated expression during the morning [44]. In rice, *SbGHD7* shows a single peak of clock-gated expression in the morning of LD [22]. It will be interesting to determine if the dual peak pattern of *PRR37* and *GHD7* expression observed in sorghum is found in other related C4 grasses such as pearl millet, Miscanthus and sugarcane.

The current study focused on characterizing the light-signaling pathway that regulates *SbPRR37* and *SbGHD7* expression in response to day length. Previous studies showed that sorghum genotypes lacking *PHYB* (58 M, *phyB-1*) flower earlier in LD compared to near isogenic genotypes (100 M) expressing *PHYB*, demonstrating that light signaling through this photoreceptor is required for photoperiod sensitive variation in flowering time [13]. The current study showed that PhyB (*Ma3*) is epistatic to genes encoding the floral repressors SbPRR37 and SbGhd7 and that PhyB is required for photoperiod-regulated expression of these genes. Moreover, 58 M, a genotype lacking functional PhyB, showed attenuated expression of *SbPRR37* and *SbGHD7* during the evening of LD compared to 100 M (PhyB). In SD, expression of the floral repressors was similar in 58 M and 100 M. Taken together, these results indicate that in sorghum PhyB is required for light signaling in LD that results in elevated expression of *SbPRR37* and *SbGHD7* during the evening.

The molecular basis of PhyB induced expression of *SbPRR37* and *SbGHD7* during the evening of long days is unknown but could involve other photoreceptors and intermediary transcription factors such as PIFs [45]. Detailed studies in rice showed that PhyA, PhyB and PhyC modulate flowering time [39]. PhyC in particular plays a role in natural variation of flowering time in pearl millet [46], Arabidopsis [47], and wheat [48]. In Arabidopsis, a long day plant, PhyB destabilizes CO, an action countered by Cry, PhyA and SPA in LD, leading to floral induction [20]. In rice, *phyB* mutants flower early in LD and SD similar to sorghum. Interestingly, rice *phyC* mutants flower early only in LD [39]. In addition, in rice, both PhyB and PhyC are required to induce *GHD7* expression, where PhyB alone causes some repression of *GHD7* mRNA levels [49]. This indicates that in rice PhyB regulates floral induction in both LD and SD, while PhyC modifies flowering time selectively in LD. The stability of PhyC is reduced in the absence of PhyB in rice and Arabidopsis [40]. PhyB increases PhyC stability, and chromophore-containing PhyB:PhyC heterodimers are required for PhyC activity [41]. Therefore, in sorghum the requirement for PhyB in photoperiod sensitive flowering time may be because PhyB increases PhyC stability and through formation of PhyB:PhyC heterodimers.

Genetic analysis of the role of *PHYB* in sorghum was examined using a population dominant for *Ma1* (*SbPRR37*) and segregating for alleles of *PHYB* (*Ma3*), *Ma5*, and *SbGHD7* (*Ma6*). The presence of *Ma1* in all progeny of the population caused delayed flowering in LD unless the expression or activity of *Ma1* (and in some genotypes *Ma1* and *Ma6*) was altered by recessive alleles of *Ma3* or *Ma5*. The analysis showed that plants homozygous for null alleles of *PHYB* (*phyB-1*) in *Ma5*_ backgrounds had reduced photoperiod sensitivity and flowered earlier in LD compared to plants encoding PhyB. Similarly, progeny homozygous for recessive alleles of *Ma5*, in *Ma3*_ backgrounds, showed reduced photoperiod sensitivity and flowered earlier in LD. The results indicated that both *PHYB* and *Ma5* are epistatic to *Ma1* and *Ma6*. Progeny recessive for either gene flowered earlier in LD, but showed a range of flowering times, indicating that other genes and/or environmental factors affected flowering time in these backgrounds, although with reduced response to photoperiod. Interestingly, *PHYB* and *Ma5* appear to be co-dependent or acting at a similar point in the regulatory pathway because allelic differences at *Ma5* did not affect flowering time significantly in *phyB-1* backgrounds and vice versa. R.07007 (*Ma3ma5*) and 58 M (*ma3^R^Ma5*) show attenuated expression of *SbPRR37* and *SbGHD7* in the evening of LD ([25] and this study) indicating that both *Ma3* (PhyB) and *Ma5* are required for elevated expression of the sorghum floral repressors during the evening of LD. In searching for an explanation for this co-dependence, we found the *Ma5* locus spans several genes known to affect flowering time including *PHYC* and that the sequence of PhyC in R.07007 (*ma5*) contained amino acid changes that could potentially modify the function of this protein. The hypothesis that *Ma5* corresponds to *PHYC* is consistent with studies showing that PhyC modifies flowering in an LD specific manner in rice, similar to *Ma5* [39]. In addition, PhyC stability is dependent in part on PhyB and PhyC activity requires the formation of functional heterodimers with PhyB (and other phytochromes) [41]. If sorghum PhyC is regulated by PhyB in a manner similar to their counterparts in rice, this would explain why *Ma5* (presumptive *PHYC*) activity is not observed in *phyB-1* backgrounds. Experiments designed to test this hypothesis are currently underway.

In Arabidopsis, *CO* expression peaks once per day in the evening and the amplitude of *CO* expression is regulated by blue light/GI-FKF1-ZTL mediated turnover of CDF1, a repressor of *CO* expression [50]. PRR7 also modifies *CO* expression through repression of *CDF1* expression [51]. In sorghum, *SbCO* expression peaks twice each day, at dawn and again in the evening in LD. The peak of *SbCO* expression at dawn is attenuated in SD ([25] and this study) and in genetic backgrounds lacking SbPRR37 [19]. It is possible that SbPRR37 modulates *SbCO* expression by repressing sorghum orthologs of *CDF1* as occurs in Arabidopsis [51]. The peak of *SbCO* expression at dawn in LD was not observed in the sorghum genotype lacking PhyB (58 M). Since PhyB is required for elevated *SbPRR37* expression in the evening of LD, and SbPRR37 has been shown to induce elevated expression of *SbCO* at dawn, it is likely that lack of PhyB induced expression of *SbPRR37* during the evenings of LD explains the observed expression of *SbCO* in 58 M.

In rice, *Hd3a*, a member of the PEBP gene family, encodes an FT protein that acts as a florigen [52]. In maize, *ZCN8* and possibly *ZCN12* are sources of florigen [24], [53]. Sorghum encodes orthologs of *Hd3a* (*SbCN15*), *ZCN8* (*SbCN8*) and *ZCN12* (*SbCN12*). *SbCN8* and *SbCN12* expression is regulated by day length and by alleles of *SbPRR37*, *SbGHD7*, and *PHYB* in a manner consistent with these genes being sources of florigen in sorghum. In prior studies, *SbCN15* expression was modulated to only a small extent by variation in photoperiod and in mutants of *SbPRR37* and *SbGHD7* that affect flowering time, suggesting that this gene was not an important target of photoperiod regulation [25], [26]. In the current study, expression of *SbCN15* was found to be ∼60-fold higher in leaves of 58 M (*phyB-1*) compared to 100 M (*PHYB*) in both LD and SD. If *SbCN15* functions as a source of florigen as in rice, photoperiod independent repression of *SbCN15* expression by PhyB suggests that this gene may be responsible for early flowering induced by shading [7]. 58 M plants exhibit shade avoidance responses including longer leaf blades and sheaths, fewer tillers, narrower leaf blades, less leaf area, and more rapid stem elongation [7]. In Arabidopsis, light signaling through PhyB represses shade avoidance responses, and PhyB deficient mutants have elongated stems and an early flowering phenotype associated with “constitutive shade avoidance” [54]. Information on photoperiod regulated flowering time in sorghum described in this paper will hopefully facilitate analysis of flowering time variation caused by shading and other environmental factors.

## Supporting Information

Figure S1 **ANOVA interaction graphs showing (A) Day-length:PhyB (Day:Genotype) interaction.** (B–D) Three two-way interactions (*Ma3*:*Ma5*, *Ma3*:*Ma6*, *Ma5*:*Ma6*) in the 58MxR.07007 F2/F3 population.
(TIF)

Figure S2 **Fold differences of** ***SbEHD1, SbCN8, SbCN12*** **and** ***SbCN15*** **RNA abundance at peaks of expression in 100 M and 58 M grown in LD (14 h light/10 h dark) or SD (10 h light/14 h dark).** Positive fold difference values indicate higher mRNA levels detected in 58 M. (A) *SbEHD1*, (B) *SbCN8*, (C) *SbCN12*, (D) *SbCN15*. The time point corresponding to peak expression is shown below each graph.
(TIF)

Figure S3 **Relative expression levels of circadian clock genes and** ***GI*** **in 100 M (black solid line) and 58 M (red dashed line) under either LD (14 h light/10 h dark) or SD (10 h light/14 h dark) conditions.** The gray shaded area represents the dark period. The first 24 h covers one light-dark cycle, followed by 24 h of continuous light. (A) GI. (B) TOC1. (C) LHY. Each data point of relative expression corresponds to three technical replicates and three biological replicates. Error bars indicates SEM.
(TIF)

Table S1 Genotypes and flowering dates of sorghum lines.
(DOCX)

Table S2 Primer sequences used for *PHYB* alleles amplification and sequencing.
(DOCX)

Table S3 Primer sequences and amplification efficiency for qRT-PCR.
(DOCX)
