# Supplementary figures and images for: Sorghum Phytochrome B Inhibits Flowering in Long Days by Activating Expression of SbPRR37 and SbGHD7, Repressors of SbEHD1, SbCN8 and SbCN12
Source: PLoS One. 2014 Aug 14;9(8):e105352. doi: 10.1371/journal.pone.0105352 (PMC4133345; doi:10.1371/journal.pone.0105352)

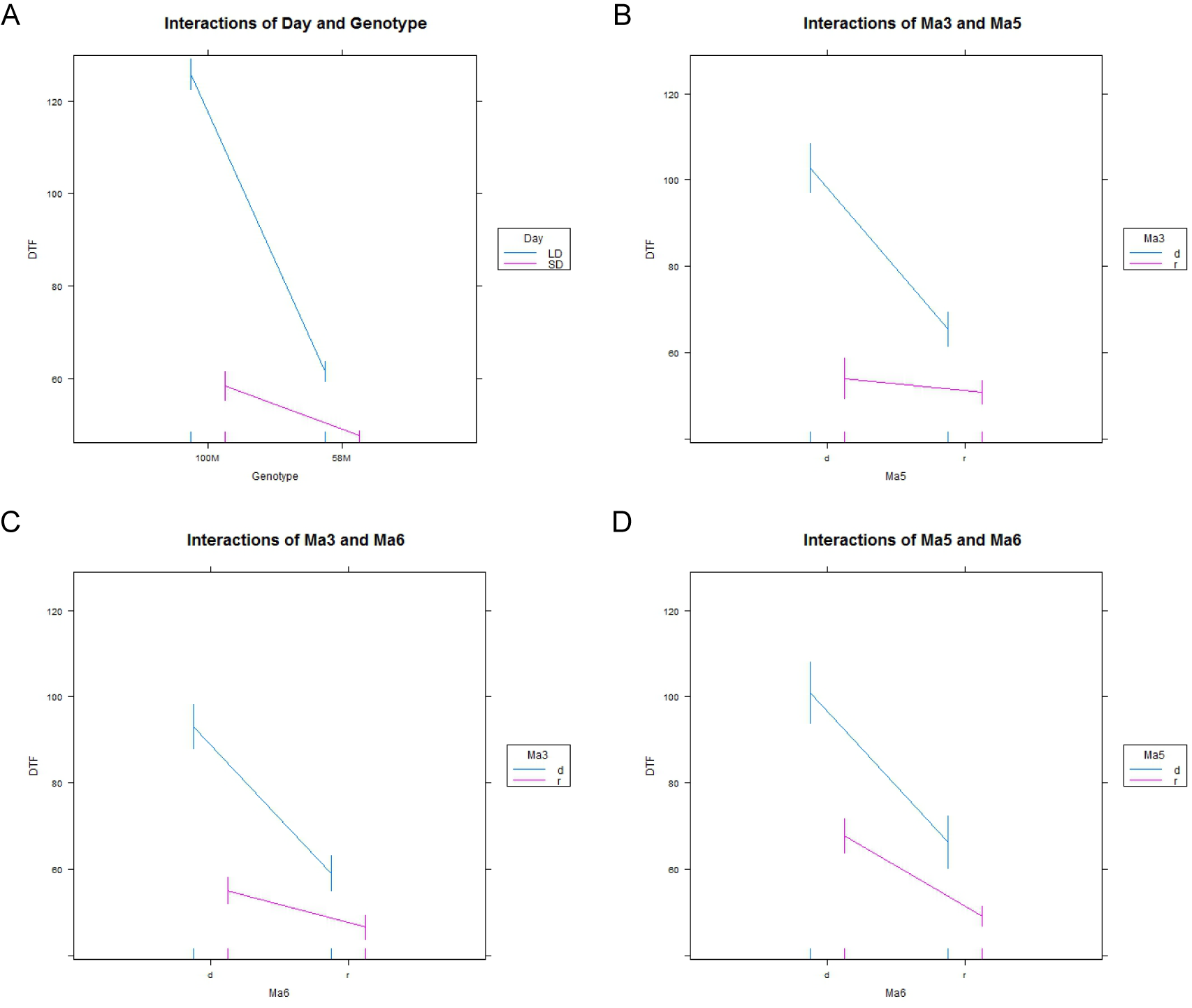

Supplement: Figure S1 — ANOVA interaction graphs showing (A) Day-length:PhyB (Day:Genotype) interaction. (B–D) Three two-way interactions (Ma3:Ma5, Ma3:Ma6, Ma5:Ma6) in the 58MxR.07007 F2/F3 population. (TIF) [file pone.0105352.s001.tif]

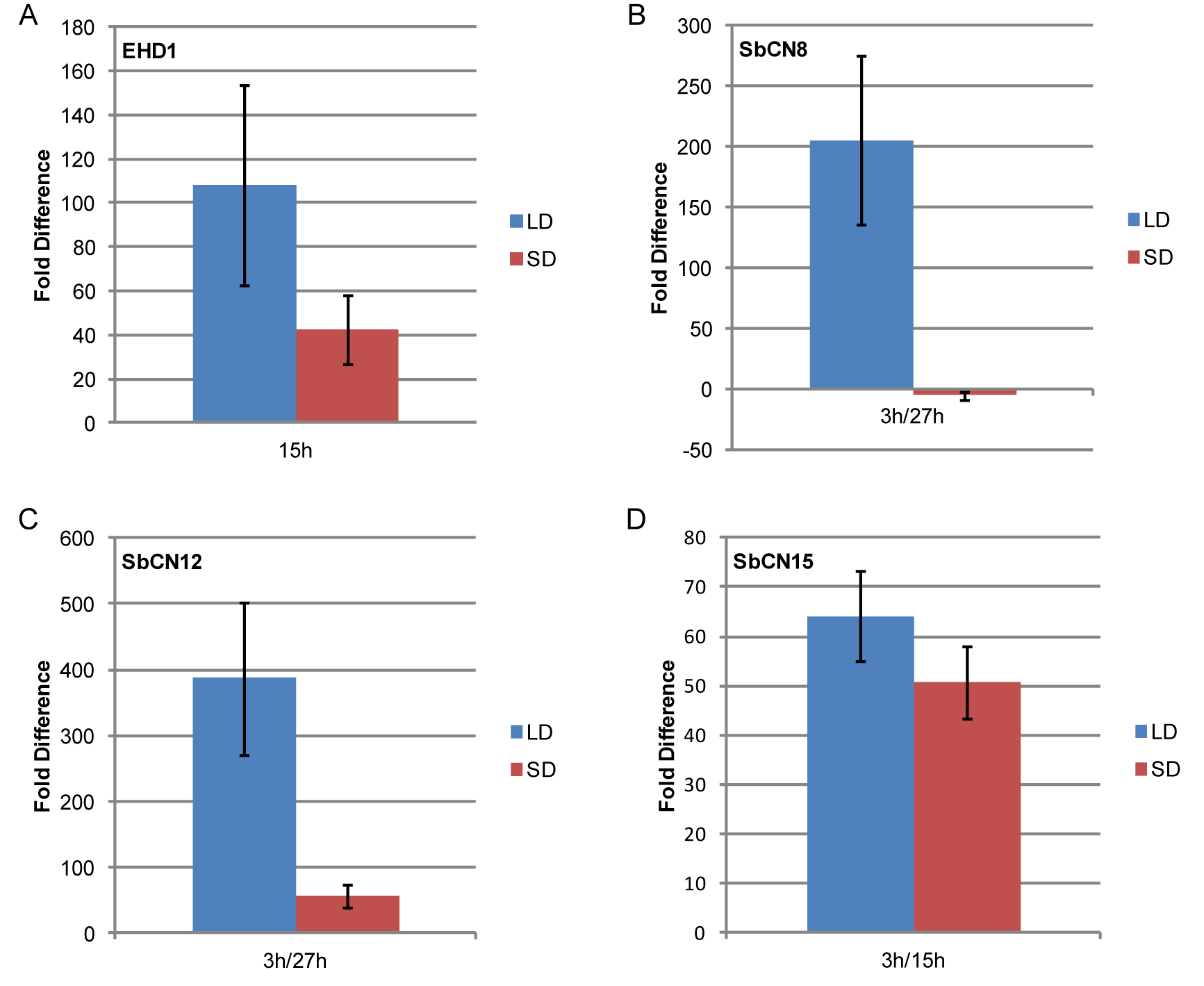

Supplement: Figure S2 — Fold differences of SbEHD1, SbCN8, SbCN12 and SbCN15 RNA abundance at peaks of expression in 100 M and 58 M grown in LD (14 h light/10 h dark) or SD (10 h light/14 h dark). Positive fold difference values indicate higher mRNA levels detected in 58 M. (A) SbEHD1, (B) SbCN8, (C) SbCN12, (D) SbCN15. The time point corresponding to peak expression is shown below each graph. (TIF) [file pone.0105352.s002.tif]

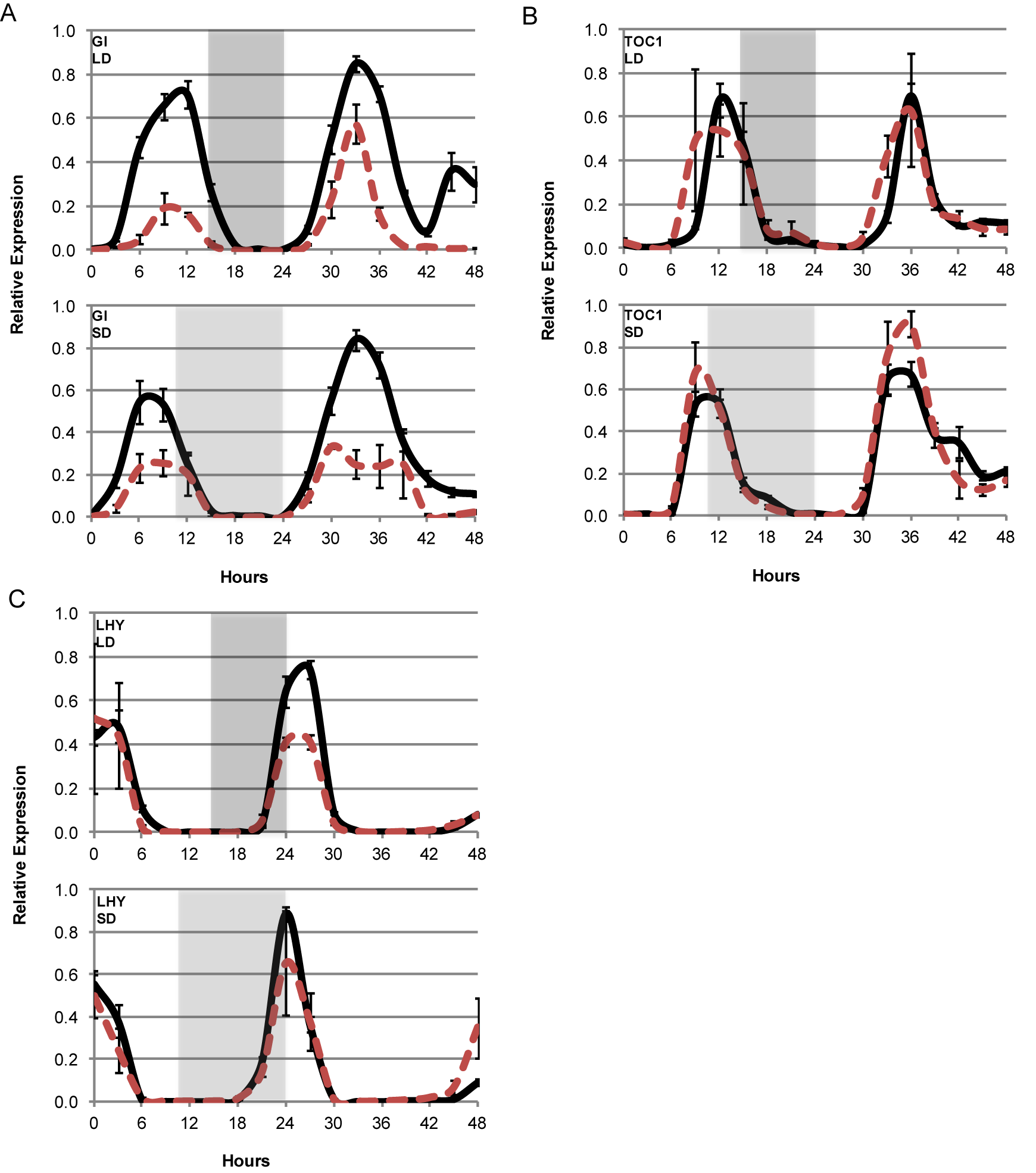

Supplement: Figure S3 — Relative expression levels of circadian clock genes and GI in 100 M (black solid line) and 58 M (red dashed line) under either LD (14 h light/10 h dark) or SD (10 h light/14 h dark) conditions. The gray shaded area represents the dark period. The first 24 h covers one light-dark cycle, followed by 24 h of continuous light. (A) GI. (B) TOC1. (C) LHY. Each data point of relative expression corresponds to three technical replicates and three biological replicates. Error bars indicates SEM. (TIF) [file pone.0105352.s003.tif]
