# Supplementary material for: Sorghum Phytochrome B Inhibits Flowering in Long Days by Activating Expression of SbPRR37 and SbGHD7, Repressors of SbEHD1, SbCN8 and SbCN12
Source: PLoS One. 2014 Aug 14;9(8):e105352. doi: 10.1371/journal.pone.0105352 (PMC4133345; doi:10.1371/journal.pone.0105352)
Supplement: Table S1 — Genotypes and flowering dates of sorghum lines. (DOCX) [file pone.0105352.s004.docx]

**Table S1. Genotypes and Flowering dates of sorghum lines.**

| **Sorghum Genotype** | **Maturity Loci** | **Days to Flowering (LD) *** |
| --- | --- | --- |
| BTx623 | *ma1Ma2Ma3Ma4Ma5ma6* | 71 |
| R.07007 | *Ma1ma2Ma3Ma4ma5Ma6* | 95 |
| F1 | *Ma1Ma2Ma3Ma4Ma5Ma6* | >160 |
| 100M | *Ma1Ma2Ma3Ma4Ma5ma6* | 126 |
| 90M | *Ma1Ma2ma3Ma4Ma5ma6* | 97 |
| 58M | *Ma1Ma2ma3^R^Ma4Ma5ma6* | 62 |

* Days to Flowering (LD) are determined in greenhouse LD (14h light/10h dark) condition.
