# Supplementary material for: Sorghum Phytochrome B Inhibits Flowering in Long Days by Activating Expression of SbPRR37 and SbGHD7, Repressors of SbEHD1, SbCN8 and SbCN12
Source: PLoS One. 2014 Aug 14;9(8):e105352. doi: 10.1371/journal.pone.0105352 (PMC4133345; doi:10.1371/journal.pone.0105352)
Supplement: Table S2 — Primer sequences used for PHYB alleles amplification and sequencing. (DOCX) [file pone.0105352.s005.docx]

**Table S2. Primer sequences used for *PHYB* alleles amplification and sequencing.**

| **Segment** | **Primer Sequence** |
| --- | --- |
| Segment 1 Amplification  (N terminal, 2190bp) | F: ATAGCCCACTTCAGCTTTCTCCCA  R: TTCTCCCATGGTAGGCTTCTGCTT |
| Segment 1 Sequencing | ATAGCCCACTTCAGCTTTCTCCCA  AGCTTCGACTACTCCCAGTC  ACACAGTCGTGGAGCATGTT  CCCCCGCCCGCGCGGGACGT  CTCGGAGGACGGCGTGGGCG  CATGACACGGTCGTAACCCG  TTCTCTGACAGCTGATGCGC  TTCTCCCATGGTAGGCTTCTGCTT |
| Segment 2 Amplification  (middle, 3693bp) | F: TTTCTCATGCAGGCATTTGGGCTG  R: AGGGATTTGAGTTTCCGTGCAAGC |
| Segment 2 Sequencing | TTTCTCATGCAGGCATTTGGGCTG  TTTTGGTTTCGGTCACACAC  CATCATCCTTATCCTCAGGG  GCTACTCTCACGAGCTTTAA  CCGGTGCTTATAACTAGCAG  TCGAGAGGTGAAGTTGTTGG  GATGATCAGAGGCAGTTCCT  TAAGGCTTGCCGCTGATACC  AAAAAAGTGCATAGTGGGGG  GCACTCCGCAATTCTCATAT  CTCCTGTGACTAGCTAACAC  GGCCCTTGAGTCGACAAAAA  CTTGGACATCTGTTTCTCAC  GTACCCAGTGCTGAGAGCA  TCAGTTCACAAGGCTATTCC  AGGGATTTGAGTTTCCGTGCAAGC |
| Segment 3 Amplification  (C terminal, 4358bp) | F: CAAGCGCTGAAATACAGCAAGCCT  R: TTAGCCAGCTTACAGCCCACCATA |
| Segment 3 Sequencing | CAAGCGCTGAAATACAGCAAGCCT  TCAAGGCTACTCCAGGCTCAAGTT  ATTGGCTGAGAGAGAAGCAGTCCA  AAAAAAGTGCATAGTGGGGG  TCAGTTCACAAGGCTATTCC  GGTTCATTGGTCCAGTGTTT  TTACTTCAACCTTTGGCTGG  CAGTATCAGAAGGCAACAGT  AGAGAGGAAAAGACCTCTGC  ACTGACCCCACCTAACTTCT  GGCTGACAACAGCATTCATA  CTTGCTTGGGACTAAAAGGC  CCAATGTGTGAGCTCAACCA  GGCTTTAACGTCGACTTTTG  TCAGGGACCTAAGTTACCTA  TTAGCCAGCTTACAGCCCACCATA |
