# Supplementary material for: Sorghum Phytochrome B Inhibits Flowering in Long Days by Activating Expression of SbPRR37 and SbGHD7, Repressors of SbEHD1, SbCN8 and SbCN12
Source: PLoS One. 2014 Aug 14;9(8):e105352. doi: 10.1371/journal.pone.0105352 (PMC4133345; doi:10.1371/journal.pone.0105352)
Supplement: Table S3 — Primer sequences and amplification efficiency for qRT-PCR. (DOCX) [file pone.0105352.s006.docx]

**Table S3. Primer sequences and amplification efficiency for qRT-PCR.**

| **Gene** | **Locus ID in Sorghum*** | **Forward Primer** | **Reverse Primer** | **Amplification Efficiency in 100M** | **Amplification Efficiency in 58M** |
| --- | --- | --- | --- | --- | --- |
| *SbPRR37* | Sb06g014570 | AACAGGACGGAACTGGAGAGAGAT | CCAAAGCAATCTTGCTAGAGGCGA | 1.16 | 1.16 |
| *SbGHD7* | Sb06g000570 | TCAGGACAACGATGACCACCAAGA | ATCAACCTCAAAGGTGAGCCCGTT | 1.19 | 1.18 |
| *SbCO* | Sb10g010050 | TAGTCCCAGACAACATGGCAACGA | AGGTCAAGTGGAGTGGCATCTGAA | 1.25 | 1.18 |
| *EHD1* | Sb01g019980 | CGTCAGGGAAGCAATGTCCTTCAT | CTTCAGTTGGAAAGCACACATCGC | 1.19 | 1.20 |
| *SbCN8* | Sb09g025760 | AACTGTCAAAGGGAAGGTGGATCG | GACTAAGCTCTCAACCCTTCAAGTC | 1.12 | 1.19 |
| *SbCN12* | Sb03g034580 | TGCATGCATGAATATCGTCGTCT | CCCGGGTAGTACATATAAGGTGGT | 1.22 | 1.16 |
| *SbCN15* | Sb10g003940 | GCTAGCTTATCCCGCATATTACCC | CCACCCAAACTGCATCCACTCTTGAA | 1.11 | 1.13 |
| *GI* | Sb03g003650 | ATGCACCCGCTTCCTAGTCATCTT | TTCAGGGCTGTCATGGTTCCTCAT | 1.15 | 1.22 |
| *TOC1* | Sb04g026190 | GAGTGCAGATGATTACTGCTCACTTTG | TGCTGCCTTGTTGCCAGTAGAAGA | 1.18 | 1.11 |
| *LHY* | Sb07g003870 | GGCCTGCCTCTACCATGAAGTTTA | GCACTGCATTGCAAGGTTTGAAGTCC | 1.15 | 1.14 |

***** Gene Locus IDs in Sorghum are based on Phytozome v8.0 (<http://www.phytozome.net/>).
